# Development of Contrast-Induced Acute Kidney Injury after Elective Contrast Media Exposure in Patients with Type 2 Diabetes Mellitus: Effect of Albuminuria

**DOI:** 10.1371/journal.pone.0106454

**Published:** 2014-09-05

**Authors:** Jun-qing Yang, Peng Ran, Ji-yan Chen, Yi-ting He, Li-wen Li, Ning Tan, Guang Li, Shuo Sun, Yong Liu, Jia-xin Zhan, Jian-yi Zheng, Ying-ling Zhou

**Affiliations:** 1 Department of Cardiology, Guangdong Cardiovascular Institute, Guangdong General Hospital, Guangdong Academy of Medical Sciences, Guangzhou, Guangdong, China; 2 Department of Cardiology, Shunde first hospital, Foshan, Guangdong, China; University of Sao Paulo Medical School, Brazil

## Abstract

**Background:**

The influence of albuminuria and urinary pH on the development of contrast-induced acute kidney disease (CI-AKI) in patients with type 2 diabetes mellitus (T2DM) after elective coronary angiography (CAG) or percutaneous coronary intervention (PCI) is unknown.

**Methods:**

CI-AKI was defined as an increase in serum creatinine >26.4 µmol/L or ≥50% of baseline value within 48 hours after contrast media exposure. Demographics, traditional risk factors, clinical outcomes and CI-AKI incidence were compared between groups. Univariate analysis and multivariate logistic regression were performed to assess risk factors of CI-AKI.

**Results:**

We observed 597 patients with T2DM after CAG or PCI. Patients were divided into 3 groups based on early morning urinary albumin: negative group (urine dipstick negative, n = 483), trace group (urine dipstick trace, n = 60), and positive group (urine dipstick ≥1+, n = 54). CI-AKI occurred in 33 (5.5%) patients, including 19 (3.9%) in the negativealbuminuria group, 4 (6.7%) in the trace group, and 10 (18.5%) in the positive group (p< 0.001), respectively. After adjusting for potential confounding risk factors, positive albuminuria (OR = 3.8, 95% CI: 1.5 to 9.2, p = 0.004) and urinary pH<6 (OR = 2.4, 95% CI: 1.1 to 5.1, p = 0.020) remained significantly associated with CI-AKI.

**Conclusion:**

Preprocedural albuminuria and urinary pH <6 are independent risk factors of CI-AKI in patients with T2DM undergoing elective cardiac catheterization, and may be used to identify patients at high risk of post-procedural CI-AKI.

## Introduction

Contrast-induced acute kidney injury (CI-AKI) is a leading cause of nosocomial acute renal failure [1], [2]. It is associated with poor outcomes, including dialysis and in-hospital [3] and 1–2 year mortality [4], [5]. Furthermore, it prolongs hospitalization and increases medical costs [6].

Albuminuria is common in patients with type 2 diabetes (T2DM). Previous studies have demonstrated that increased albuminuria and reduced estimated glomerular filtration rate (eGFR) are independent risk factors for acute kidney injury [7], and cardiovascular and renal events [8] in T2DM patients. However, the impact of albuminuria on the prevalence of CI-AKI in T2DM patients is unclear, and few studies have investigated the relationship between urinary albumin levels and CI-AKI. In addition, the pathogenesis of CI-AKI may include free radicals production, which is promoted by the acidic environment in the urinary tubule [9]. Urinary pH may be associated with CI-AKI.

Therefore, we investigated influence of urinary albumin levels and urinary pH on the incidence of CI-AKI in T2DM patients who underwent elective CAG or PCI.

## Methods

### Study protocol

We conducted an observational study in consecutive patients with T2MD and who underwent elective CAG or PCI at the Guangdong Cardiovascular Institute of Guangdong General Hospital, Guangdong Academy of Medical Sciences, between August 2009 and August 2012. The study protocol was approved by the Guangdong General Hospital ethics committee, and all patients provided a written informed consent.

T2DM patients undergoing CAG or PCI were enrolled. Exclusion criteria were: 1) age <18years; 2) eGFR<15 ml/min/1.73 m^2^; 3) dialysis; 4) nephrotic syndrome; 5) nephropyelitis; 6) emergency CAG or PCI; 7) cardiac shock; 8) severe aortic valve disease; 9) multiple myeloma; 10) severe hepatic insufficiency; 11) acute stroke; 12) diabetic ketoacidosis; 13) iodic contrast administration during the preceding 7 days; 14) coronary revascularization surgery; 15) nonsteroidal anti-inflammatory drugs (NSAIDs) use within 48 hours before the procedure; 16) severe sepsis; 17) pregnancy; 18) allergy to contrast medium.

Routine urinalysis of early morning urine samples which involved urinary albumin and pH was performed upon hospital admission and before the procedure. Serum creatinine was measured as part of routine clinical care upon admission, and daily for 2 days after the procedure. Urinary albumin and pH were tested using an Urisys-2400 automatic analyzer (Roche Diagnostics, Basel, Switzerland), a Miditrom Junior analyzer (Roche Diagnostics, Basel, Switzerland), or a COBAS U411 analyzer (Roche Diagnostics, Basel, Switzerland). eGFR was estimated using the abbreviated Modification of Diet in Renal Disease (MDRD) formula [10].

CAG or PCI was performed using standard techniques [11]. The dose and types of contrast medium were left to the discretion of the interventional cardiologist. The use of aspirin, clopidogrel, β-blockers, angiotensin-converting enzyme inhibitors (ACEIs)/angiotensin receptor blockers (ARBs), calcium channel blockers (CCBs), diuretics, intra-aortic balloon pump (IABP) or vascular active drugs was also left to the discretion of the interventional and/or clinical cardiologist. Patients received perioperative hydration for 8–36 hours with normal saline at a rate of 1 ml/kg/hour. The hydration rate was reduced by half in patients with a left ventricular ejection fraction (LVEF) <35% or NYHA class III–IV at admission.

T2DM was defined as fasting plasma glucose ≥126 mg/dl (7.0 mmol/L); or 2-h plasma glucose ≥200 mg/dl (11.1 mmol/L) during an oral glucose tolerance test; or in a patient with classic symptoms of hyperglycemia or hyperglycemic crisis, a random plasma glucose ≥200 mg/dl (11.1 mmol/L) [12], a previous diagnosis or received treatment was also accepted. Anemia was defined as baseline hematocrit <39% for men and <36% for women. Hypotension was defined as systolic blood pressure (SBP) <80 mmHg for at least 1 hour requiring inotropic support with medications or intra-aortic balloon pump (IABP) within 24 hours periprocedurally. Congestive heart failure was defined as New York Heart Association functional classification III/IV and/or history of pulmonary edema [13].

### Endpoints

#### Primary endpoints

The primary endpoint was the occurrence of CI-AKI, was defined as an increase in serum creatinine >26.4 µmol/L or ≥50% of baseline value within 48 hours after contrast media exposure [14].

#### Secondary endpoints

Secondary endpoints were absolute change in serum creatinine within 48 hours, in-hospital death and dialysis. The cause of death was ascertained from discharge documents and discussion with attending physician.

### Follow-up

Follow-up was performed at an outpatient clinic visit or via telephone interviews. Cardiac death was defined as any death with a demonstrable cardiac cause or any death that was not clearly attributable to a non-cardiac cause [15]. All-cause death, cardiac death, and dialysis were adjudicated by two experienced cardiologists who were blinded to angiographic and biochemical data.

### Statistical analysis

SPSS 19.0 was used for statistical analysis. Patients were divided into three groups according to early morning urinary albumin assessment by routine urinalysis: negative group (urine dipstick negative), trace group (urine dipstick trace), and positive group (urine dipstick ≥1+). Data are presented as mean ± standard deviation (SD) for normally distributed data, or medians and interquartile ranges (IQR) for unevenly distributed data. Categorical data are expressed as numbers (%). Demographics, traditional risk factors, clinical outcomes and CI-AKI incidence were compared between groups. Chi-square tests or Fisher exact tests were used for categorical data, as appropriate. Normally distributed variables were compared by one-way ANOVA (with Bonferroni correction for comparisons between groups if the ANOVA p-value was <0.05), or by the Kruskal-Wallis test for unevenly distributed data.Risk factors were initially screened for univariate association with CI-AKI at a p value <0.20, and identified variables were then assessed in a forward stepwise manner using a p-value criterion of <0.05. The final model included the important baseline characteristics. Multivariate logistic regression analysis was performed to identify independent risk factors for CI-AKI. Cumulative event curves were constructed using the Kaplan-Meier survival method, and cumulative event rates were compared using the log-rank test. All p-values were two-tailed, and a p-value <0.05 was considered statistically significant.

## Results

A total of 597 patients with T2DM after elective CAG or PCI were included; 173 (29.0%) were female, and the overall patient age was 65.1±10.1 years. Baseline patient eGFR and serum creatinine were were 77.8 ml/min/1.73 m^2^ (IQR 61.6–96.7 ml/min/1.73 m^2^) and 84.0 µmol/L (IQR 70.5–106.0 µmol/L), respectively. Overall, 33 patients (5.5%) developed CI-AKI

### Baseline characteristics

Table 1 shows the baseline characteristics of the study population. There were significant differences among negative, trace and positive groups including: serum creatinine { 82.0(IQR 68.0–101.0)µmol/L vs. 94.4(IQR 75.3–113.5)µmol/L vs. 117.6(IQR 95.3–190.0)µmol/L, p<0.001}, urea nitrogen {4.8(IQR 3.8–6.2) mmol/L vs. 5.4(IQR 4.4–7.0)mmol/L vs.7.4(IQR 5.0–9.8) mmol/L, p<0.001}, eGFR { 80.2 (IQR 64.0–98.9)ml/min/1.73 m^2^ vs.72.3(IQR 50.6–97.3)ml/min/1.73 m^2^ vs.54.5(IQR 31.1–74.8) ml/min/1.73 m^2^, p<0.001}, the proportion of preoperative congestive heart failure (13.0% vs.16.7% vs. 25.9%, p = 0.011), anemia (36.5% vs.40.0% vs. 66.7%,p<0.001), hypertension (67.9% vs.75.0% vs. 88.9%, p = 0.001), hypoalbuminemia (36.2±4.6 g/L vs. 33.7±4.3 g/L vs. 31.3±4.9 g/L, p<0.001), SBP(133.2±20.0 mmHg vs. 137.6±20.1 mmHg vs. 141.8±27.1 mmHg, p = 0.002), HbA1c (7.6±1.5% vs. 8.5±2.2% vs. 7.8±1.6, p = 0.014), and use of diuretics (13.9% vs.18.3% vs. 38.9%, p<0.001). There was no difference in age, LVEF, periprocedural hypotension, periprocedural IABP, urinary pH, history of myocardial infarction, or hyperlipidemia.

**Table 1:** Baseline Characteristics.

characteristics	Urinary Albumin				*p*			
	All (N = 597)	Negative (N = 483)	Trace (N = 60)	Positive (N = 54)	All	Negative vs. Trace	Trace vs. Positive	Positive vs. Negative
Female, n(%)	173(29.0%)	151(31.3%)	9(15.0%)	13(24.1%)	0.046	-	-	-
Age, years	65.1±10.1	65.0±10.1	64.7±10.0	66.5±9.5	0.541	-	-	-
eGFR,	77.8	80.2	72.3	54.5	<0.001^*^	-	-	-
ml/min/1.73 m^2^	(61.6-96.7)	(64.0-98.9)	(50.6-97.3)	(31.1-74.8)				
SBP, mmHg	134.4±21.0	133.2±20.0	137.6±20.6	141.8±27.1	0.002	0.378^#^	0.838^#^	0.012^#^
DBP, mmHg	77.1±11.9	77.1±11.9	76.6±10.8	78.0±13.5	0.737	-	-	-
HR, bpm	74.7±11.7	74.6±11.3	75.1±11.4	75.7±14.1	0.461	-	-	-
LVEF, %	59.2±12.8	59.7±12.7	57.2±13.4	57.4±12.5	0.120	-	-	-
Hypotension, n(%)	14(2.3%)	12(2.5%)	1(1.7%)	1(1.9%)	0.682	-	-	-
IABP, n(%)	10(1.7%)	7(1.4%)	1(1.7%)	2(3.7%)	0.260	-	-	-
Congestive heart failure, n(%)	87(14.6%)	63(13.0%)	10 (16.7%)	14(25.9%)	0.011	-	-	-
**Medical history**								
Smoking, n(%)	20(34.3%)	162(33.5%)	28(46.7%)	15(27.8%)	0.965	-	-	-
Hypertension, n(%)	421(70.5%)	328(67. 9%)	45(75.0%)	48(88.9%)	0.001	-	-	-
Dyslipidemia, n(%)	82(13.7%)	69(14.3%)	6(10.0%)	7(13.0%)	0.555	-	-	-
Anemia, n(%)	235(39.6%)	175(36.5%)	24(40.0%)	36(66.7%)	<0.001	-	-	-
MI history, n(%)	87(14.6%)	66(13.7%)	8(13.3%)	13(24.1%)	0.075	-	-	-
CABG history, n(%)	7(1.2%)	6(1.2%)	1(1.7%)	0(0.0%)	0.552	-	-	-
**Laboratory measurements**								
SCr, µmol/L	84.0 (70.5-106.0)	82.0 (68.0–101.0)	94.4 (75.3–113.5)	117.6 (93.5–190.0)	<0.001^*^	-	-	-
BUN, mmol/L	5(4.0–6.4)	4.8(3.8–6.2)	5.4(4.4–7.0)	7.4(5.0–9.8)	<0.001^*^	-	-	-
TC, mmol/L	4.3±1.1	4.2±1.1	4.2±1.0	4.5±1.1	0.211	-	-	-
LDL-C, mmol/L	2.4(1.9–3.0)	2.4(1.9–3.0)	2.3(1.8–3.2)	2.5(2.0–3.1)	0.871^*^	-	-	-
TG, mmol/L	1.4(1.0–1.9)	1.4(1.0–1.9)	1.5(1.0–1.8)	1.5(1.2–2.6)	0.158^*^	–	-	-
HbA1c, %	7.7±1.6	7.6±1.5	8.5±2.2	7.8±1.6	0.014	<0.001^#^	0.029^#^	1.000^#^
Urinary PH<6,n(%)	179(30.0%)	140(29.0%)	20(33.3%)	19(35.2%)	0.271	-	-	-
PH<5.5	155	27	13	15				
PH = 5.5	24	13	7	4				
Hematocrit (%)	38.0±4.8	38.0±4.6	39.0±4.4	35.0±6.2	<0.001	1.000^#^	0.002^#^	<0.001^#^
Hemoglobin, g/L	129.2±16.5	130.22±15.4	132.0±16.2	116.8±20.6	<0.001	1.000^#^	<0.001^#^	<0.001^#^
Albumin, g/L	35.5±4.8	36.2±4.6	33.7±4.3	31.3±4.9	<0.001	0.049^#^	0.097^#^	<0.001^#^
**Medicine usage**								
ACEI/ARB, n (%)	531(88.9%)	434(89.9%)	49(81.7%)	48(88.9%)	0.351	-	-	-
β-blocker, n (%)	515(86.3%)	421(87.2%)	52(86.7%)	42(77.8%)	0.087	-	-	-
CCB, n (%)	156(26.2%)	116(24.1%)	18(30.0%)	22(40.7%)	0.007	-	-	-
Diuretics, n (%)	99(16.6%)	67(13.9%)	11(18.3%)	21(38.9%)	<0.001	-	-	-
Statin, n (%)	584(97.8%)	473(97.9%)	60(100.0%)	51(94.4%)	0.289	-	-	-
Insulin, n (%)	175 (29.3%)	125 (25.9%)	26 (43.3%)	24(44.4%)	<0.001	-	-	-
α-glycosidase inhibitors, n (%)	341(57.1%)	284(58.8%)	33(55.0%)	24(44.4%)	0.046	-	-	-
Sulfonylureas, n(%)	169(28.3%)	147(30.4%)	17(28.3%)	5(9.3%)	0.003	-	-	-
Repaglinide/Nateglinide, n(%)	80 (13.4%)	67 (13.9%)	8 (13.3%)	5 (9.3%)	0.381	-	-	-
Thiazolidinediones, n(%)	13(2.2%)	11(2.3%)	2 (3.3%)	0 (0%)	0.453	-	-	-

1. Data are expressed as number (%), or mean±SD for normally distributed data, or median (IQR) for unevenly distributed data.

2.^*^ P was tested by Kruskal-Wallis test.

3. ^#^ P was tested by Bonferroni correction.

4. Chi-square test or Fisher exact test was used for the frequency of distribution. Means were compared by One-way ANOVA for normally distributed data.

5. SBP: Systolic blood pressure. DBP: diastolic blood pressure. BUN: blood urea nitrogen. LVEF: left ventricular ejection fraction; ACEI/ARB: angiotensin-converting enzyme inhibitor/angiotensin receptor blocker. CABG: coronary artery bypass grafting. CCB: Calcium channel blocker. MI: myocardial infarction. HbA1C: glycosylated hemoglobin A1c. LDL-C: low density lipoprotein-cholesterol. TG: Triglycerides.

### Procedural characteristics

Table 2 lists the procedural characteristics of the patients. Compared with the negative and trace groups, the patients in the positive urine albumin group were hydrated with higher volumes of fluid (500 ml (IQR 500–1000 ml) and 500 ml (IQR 500–1000 ml) vs. 1000 ml (IQR 500–1500 ml); p = 0.002), presumably because patients with albuminuria presented with reduced baseline renal function and underwent longer hydration. There were no differences in the other procedural characteristics.

**Table 2:** Procedural Characteristics.

Characteristics	Urinary Albumin	*p*
	All (N = 597)	Negative (N = 483)	Trace (N = 60)	Positive (N = 54)	
Number of lesions (n)	2.1±1.1	2.0±1.1	2.0±1.0	2.3±1.1	0.322
Number of stents (n)	1.6±1.4	1.5±1.4	1.6±1.4	1.6±1.2	0.945
Left main lesion (%)	64(10.7%)	54(11.2%)	5(8.3%)	5(9.3%)	0.520
Left main treated (%)	30(5.0%)	28(5.8%)	1(1.7%)	1(1.9%)	0.100
Total stent length (mm)	38.4±36.6	38.1±37.2	40.3±36.4	38.7±32.4	0.912
Contrast Type					0.263
Iopamidol	328(55.1%)	265(55.0%)	33(55.0%)	30(56.6%)	
Iopromide	238(40.0%)	199(41.3%)	23(38.3%)	16(30.2%)	
Iodixanol	25(4.2%)	16(3.3%)	3 (5.6%)	6 (11.3%)	
Contrast volume, ml	130 (80–170)	130 (80–165)	130 (100–150)	135 (77.5–172.5)	0.901^*^
Intravenous hydration volume, ml	500 (500–1000)	500 (500–1000)	500 (500–1000)	1000(500–1500)	0.002^*^

1. Data are expressed as number (%), or mean±SD for normally distributed data, or median (IQR) for unevenly distributed data.

2. ^*^P was tested by Kruskal-Wallis test.

3. PCI: percutaneous coronary intervention.

### CI-AKI incidence and in-hospital outcomes

CI-AKI incidence and in-hospital clinical outcomes are displayed in Table 3. The CI-AKI incidence in positive urine albumin group was higher than the negative and trace groups (negative vs. trace vs. positive: 3.9% vs.6.7% vs. 18.5%, p<0.001). A difference was observed in dialysis frequency (0.2% vs. 1.7% vs. 1.9%, p = 0.005). No difference was observed regarding in-hospital death.

**Table 3:** CI-AKI incidence and Clinical outcomes in-hospital.

Outcomes	Urinary Albumin				*p*
	All (N = 597)	Negative (N = 483)	Trace (N = 60)	Positive (N = 54)	
CI-AKI					
SCr increase≥26.4µmol/L or ≥50%, n(%)	33(5.5%)	19(3.9%)	4(6.7%)	10(18.5%)	<0.001
SCr increase≥26.4µmol/L, n(%)	31(5.2%)	18(3.7%)	3(5.0%)	10(18.5%)	<0.001
SCr increase≥50%, n(%)	10(1.7%)	6(1.2%)	2(3.3%)	2 (3.7%)	0.101
Absolute change in SCr, µmol/L	1.3(−5.8–10.0)	0.9(−5.5–9.0)	2.3(−6.0–10.0)	10.1(−7.8–20.1)	0.016*
Death, n(%)	3 (0.5%)	1(0.2%)	2(3.3%)	0(0%)	0.280
Dialysis, n(%)	2(0.5%)	0(0%)	1(1.7%)	1(1.9%)	0.005

CI-AKI: contrast-induced acute kidney injury; SCr: serum creatinine;

^*^ P was tested by Kruskal-Wallis test.

### Multivariate analysis

Results of multivariate logistic regression analysis have been displayed in Table 4. After adjusting for potential confounding risk factors (Hosmer-Lemeshow Chi-square: 5.2, p = 0.520), and using the negative group as reference, the odds ratio (OR) of CI-AKI was 3.8 (95% CI: 1.5–9.2, p = 0.004) for the positive urine albumin group, but 1.4 (95%CI: 0.4–4.5, p = 0.562) for the trace urine albumin group. The OR of CI-AKI was 2.4 (95% CI: 1.1–5.1, p = 0.020) for urinary pH <6, and 2.5 (95% CI: 1.1–5.7, p = 0.023) for eGFR <60 ml/min/1.73 m^2^. However, age >65 years and congestive heart failure were not associated with CI-AKI in this study.

**Table 4:** Univariate and multivariate analyses of predictors for CI-AKI.

Risk factors	Univariate analysis			Multivariate analysis		
	OR	95%CI	*p*	OR	95%CI	*p*
Urinary Albumin			<0.001			0.015
Negative	-	-	-	-	-	-
Trace (vs. Negative)	1.7	0.6–5.3	0.327	1.4	0.4–4.5	0.562
Positive (vs. Negative)	5.6	2.4–12.7	<0.001	3.8	1.5–9.2	0.004
Urinary PH<6.0	3.0	1.5–6.1	0.002	2.4	1.1–5.1	0.020
eGFR<60 ml/min/1.73 m^2^	5.0	2.4–10.3	<0.001	2.5	1.1–5.7	0.023
Age>65 years	2.6	1.2–5.8	0.016	1.8	0.8–4.1	0.191
Congestive heart failure*	3.7	1.8–7.9	0.001	2.1	0.9–4.8	0.074

^*^Congestive heart failure was defined as New York Heart Association functional classification III/IV and/or history of pulmonary edema.

Hosmer-Lemeshow Chi-square: 5.2, *p* = 0.520.

### Follow-up

Median follow-up was 23 months (IQR: 16 to 30 months). Clinical outcomes were available for 572 patients (95.8%). Forty-two (7.5%) patients died. Cardiac death occurred in 11 patients in the positive urine albumin group due to refractory heart failure (n = 6), acute myocardial infarction (n = 1), cardiac arrest (n = 1), or other cardiovascular causes (n = 3). In the trace urine albumin group, 4 cardiac deaths were caused by refractory heart failure (n = 1), acute myocardial infarction (n = 1), cardiac arrest (n = 1), or other cardiovascular causes (n = 1). In the negative urine albumin group, 16 cardiac deaths were attributed to refractory heart failure (n = 6), acute myocardial infarction (n = 6), sudden death (n = 2), or other cardiovascular causes (n = 2).

**Figure 1:**
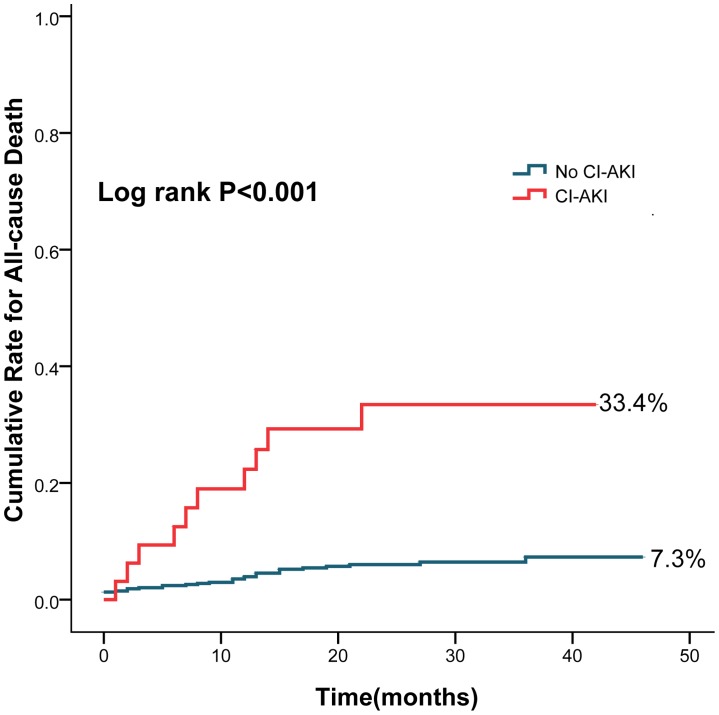
Cumulative rates for all-cause death in patients with CI-AKI and without CI-AKI.

**Figure 2:**
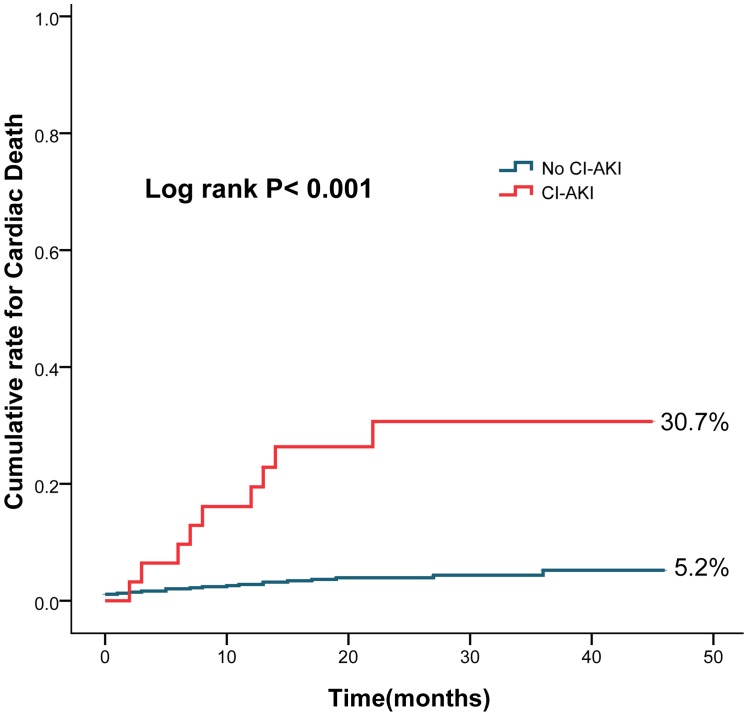
Cumulative rates for cardiac death in patients with CI-AKI and without CI-AKI.

**Figure 3:**
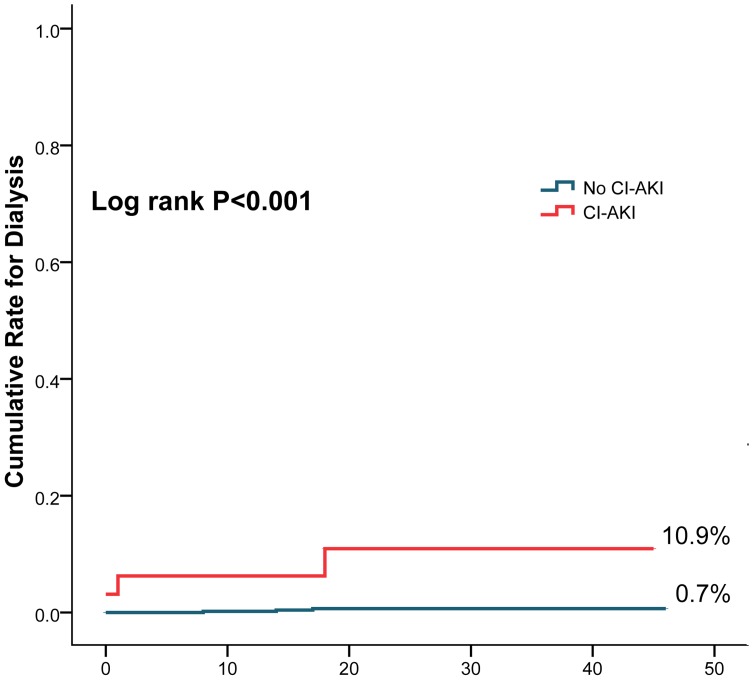
Cumulative rates for dialysis in patients with CI-AKI and without CI-AKI.

The cumulative rate of all-cause death (33.4% vs. 7.3%, log rank P<0.001) (Fig.1), cardiac death (30.7% vs. 5.2%, log rank P<0.001) (Fig.2) and dialysis (10.9% vs. 0.7%, log rank P<0.001)(Fig.3) were higher in patients with CI-AKI compared with those without.

## Discussion

The present study suggests that pre-procedural urinary albumin levels, urinary pH <6 and eGFR <60 ml/min/1.73 m^2^ were independent predictors for CI-AKI in patients with T2DM undergoing elective cardiac catheterization (CAG or PCI).

The mechanism of CI-AKI/contrast-induced nephropathy (CIN) is not clear. However, it probably involves the direct toxic effect of contrast agents and decreased renal medullary blood flow resulting in medullary ischemia that may lead to enhanced ROS formation and oxidative stress.

Many studies have indicated that preexisting renal disease [13], [16], [17] and T2DM [13], [16]–[20] are risk factors for CI-AKI/CIN. In the Mehran's study, preexisting renal disease including eGFR <60 ml/min/1.73 m^2^ or serum creatinine >1.5 mg/dl, is an independent predictor of CIN [13]. European Society of Urogenital Radiology (ESUR) contrast media safety Committee [14] considers that serum creatinine is not an ideal marker for renal function, and support eGFR to identify impaired renal function. In present study, eGFR <60 ml/min/1.73 m^2^ was an independent risk factors for CI-AKI (OR = 2.5, 95% CI: 1.1–5.7, P = 0.023). The possible reason is that preexisting renal disease is associated with a decreased vasodilatory response and slower clearance of contrast media compared to normal subjects. Besides, in the Mehran's study [13], T2DM is an independent predictor of CIN. The possible mechanisms are: impaired endothelial function, caused by persistent hyperglycemia [21], leading to impaired renal vasomotor function and renal ischemia; and increased ROS generationas well as preexisting renal failure or metabolic syndrome [17].

Albuminuria may be an additional risk factor for T2DM patients. Albumin is almost totally restricted by the glomerular barrier, filtered albumin is reabsorbed by the proximal tubular cells [22], degraded there, and brings about inflammatory and fibrogenic mediators resulting in inflammation and fibrosis, followed by loss of renal function [22], [23]. Albuminuria may contribute to the pathogenesis of CI-AKI/CIN by its toxic effects on the tubular system [24]. Albuminuria could activate Fas-mediated and peroxisome proliferator-activated receptor-γ-dependent apoptosis, and induce proinflammatory molecules, leading to apoptosis and damage of the renal tubular system [25]–[32]. In addition, albumin overload in proximal tubules could induce ROS formation, which activates NF-κB and consequently evokes NF-κB-dependent inflammatory reactions [33]. Moreover, in animal models of massive proteinuria, excessive oxidative stress induced by oxidized fatty acid was noted in regions with renal tubular damage [34]. Albuminuria and contrast media may share similar effects on kidneys [24], and may coordinate with each other in the pathogenesis of CI-AKI/CIN.

James et al. [7] demonstrated that the incidence of AKI is about 4 times higher in patients with heavy albuminuria (relative risk: 4.4 vs. no albuminuria, 95% CI: 3.7–5.2). Furthermore, long-term outcomes such as dialysis, death and composite renal outcomes are worse in patients with heavy albuminuria. In other studies [8], [35]–[38], similar conclusions were reached, i.e. that albuminuria was an independent risk factor for AKI, renal and cardiovascular events, and all-cause mortality. In addition, albuminuria and eGFR were independently associated with AKI [39]. Chronic kidney disease (CKD) is defined as either kidney damage (proteinuria, hematuria or anatomical abnormality) or eGFR<60 ml/min/1.73 m^2^ present on at least 2 occasions for ≥3 months [40]. The diagnosis of proteinuria supports the diagnosis of CKD in T2DM patients, independently of eGFR values [41]. Albuminuria may represent another type of CKD. Albuminuria may be an independent predictor for CI-AKI. A recent study [24] concluded that proteinuria may be a new risk factor for CIN in CKD patients; however, the sample size was small. Our data supported the hypothesis that urinary albumin levels were associated with CI-AKI incidence, and that albuminuria was an independent risk factor for CI-AKI in patients with T2DM.

ROS formation plays an important role in the development of CI-AKI/CIN [42] and may be promoted by an acidic urinary environment, but is inhibited by higher pH [22], [39], and alkalinizing renal tubular fluid with bicarbonate may reduce injury. As with others, this is the most important mechanism why sodium bicarbonate provided more protection than sodium chloride in animal models for acute ischemic renal failure [43], clinical trials [9], [44], [45] and meta analyses [46]–[48] for CIN. Besides, sodium bicarbonate appears capable to scavenge ROS, and increasing urine flow. Additional, sodium bicarbonate avoids the large amounts of chloride which is in isotonic saline that may cause constriction of the renal vasculature. Therefore, guidelines by ESUR contrast media safety Committee considers that sodium bicarbonate seems to provide equal or superior protection to isotonic saline [14]. Actually, urinary pH is used in the diagnosis of renal tubular acidosis (RTA), despite its low specificity. Urinary pH could partly indicate renal tubular fluid pH, and a low urinary pH (<5.5) could be found in various forms of RTA (mostly in types 2 and 4 RTA) [49], [50]. In the present study, among patients with urinary pH <6 most (n = 155, 86.59%) were of <5.5, and some may be with RTA. In a recent study[51], CIN incidence was significantly lower in patients with urinary pH >6 (32.8% vs. 2.2% in those with urinary pH >6, P<0.0001). The study by Merten et al. [9] showed that lower post-procedure urinary pH (5.6±0.6 vs.6.5±0.8, P = 0.002) and lower CIN incidence in the group hydrated by sodium bicarbonate compared with sodium chloride. Despite a small sample size (n = 119) and a weak statistical power [52], patients with urinary pH <6 may be a population that could benefit from bicarbonate hydration to prevent CI-AKI/CIN. Another study [53] concluded that daily oral sodium bicarbonate could slow eGFR decline and reduce urinary albumin based on a 5-year follow-up.

Albuminuria and urinary pH <6 are valuable for identifying patients at high risk of CI-AKI. What adds to the value of these parameters is that they are included in the routine test for every patient, and easy to obtain from preoperative urinalysis. Using these existing data, we may assess every patient more accurately and give him/her more precise risk stratification, importantly, with no additional cost and little effort.

### Limitations

Our study has several limitations. First, this study was observational, of moderate-scale, and from a single center. Some patients were excluded such as patients with a nephrotic syndrome. The results should be confirmed in a large-scale multicenter clinical trial, including patients excluded from the current analysis. Second, treatment-related details in coronary artery disease were not available. Available information was restricted to cardiovascular drugs used. Finally, the data of some useful biochemical parameters such as cystatin C and urinary albumin/creatinine ratio were lacking in the present study.

## Conclusions

Preprocedural albuminuria and urinary pH <6 were independent factors for CI-AKI in T2DM patients undergoing elective cardiac catheterization. Urinary albumin levels and urinary pH may be potential biochemical parameters for screening high-risk patients.
